# Supplementary material for: Faster bi-stable visual switching in psychosis
Source: Transl Psychiatry. 2024 May 7;14:201. doi: 10.1038/s41398-024-02913-z (PMC11076514; doi:10.1038/s41398-024-02913-z)
Supplement: Supplementary file 1 — Supplementary Figs. [file 41398_2024_2913_MOESM1_ESM.pdf]

Supplemental figures

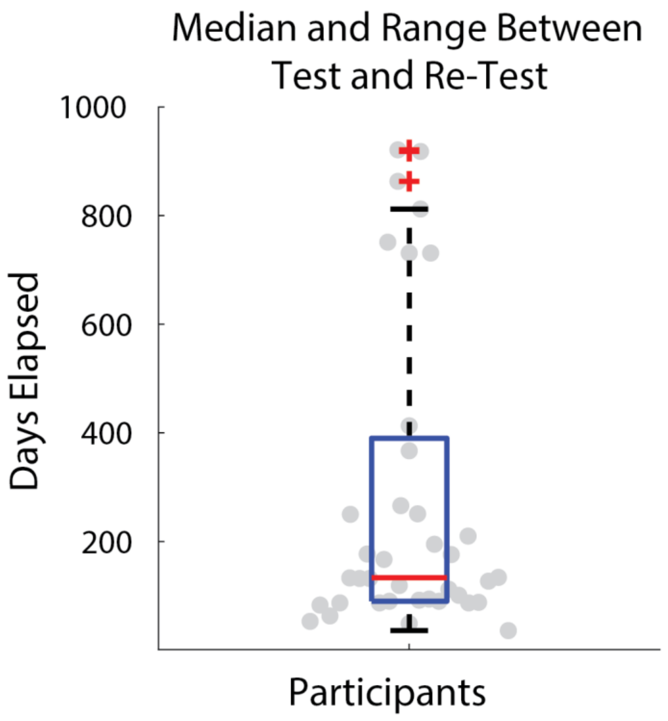

Supplemental Figure 1. Median and range of days between test and re-test sessions. Box plot of 1.5 x the interquartile range (dashed line), 25% - 75% quartiles (blue), and median (red) of the number of days between the first and second sessions for all participants, who took part in both sessions. Outliers are shown with red crosses.

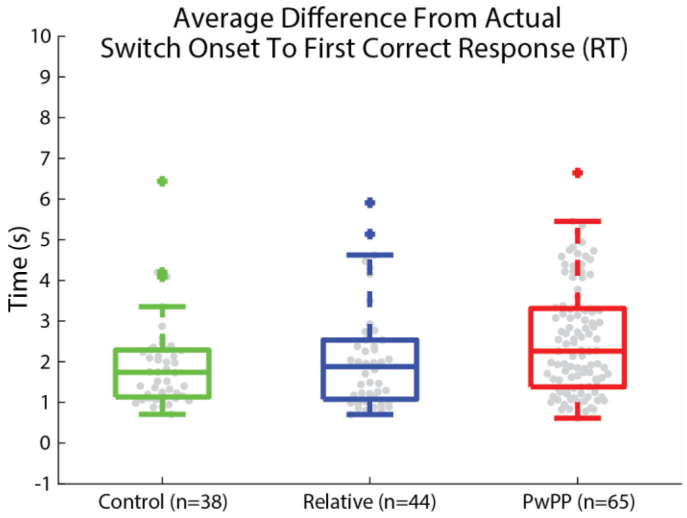

Supplemental Figure 2. Average difference across groups between the physical switch onset and the first correct response. Boxplots show median (middle line) 25-75% (box), 1.5 x the interquartile range (whiskers), and outliers (pluses), as well as individual data points (gray).

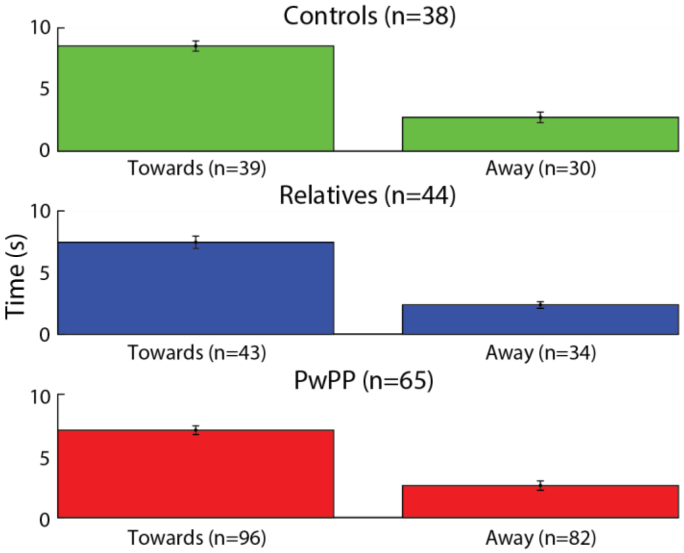

Supplemental Figure 3. Average percept duration for responses made toward or away from the direction of the physical switch. Plotted is the average time between reporting a switch and reporting the next switch (either away from or towards the actual physical rotation direction), for each group. Error bars represent the standard error of the mean.

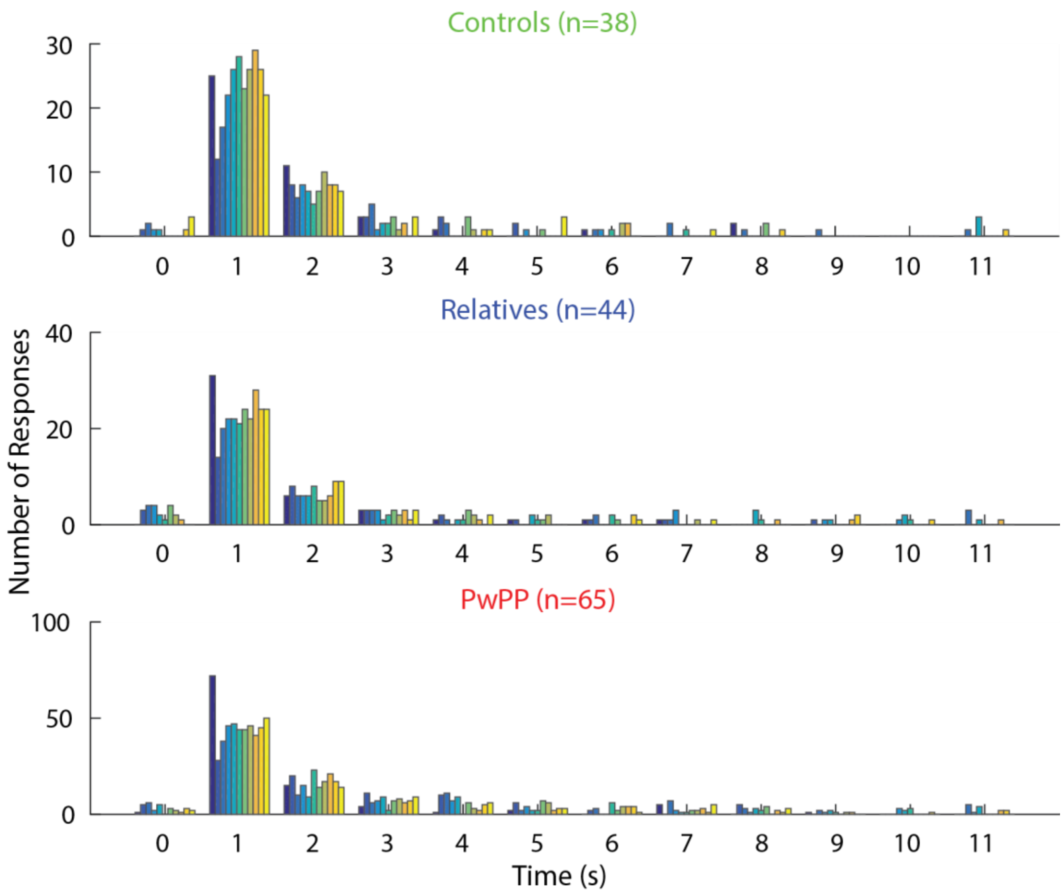

Supplemental Figure 4. Distribution of total responses made by response time. Plotted are the number of responses (y-axis) made within each time bin (x-axis) for 11 s following a stimulus direction change. Colored bars represent physical switch number (11 total physical switches per subject) ordered from first to last presented switch.

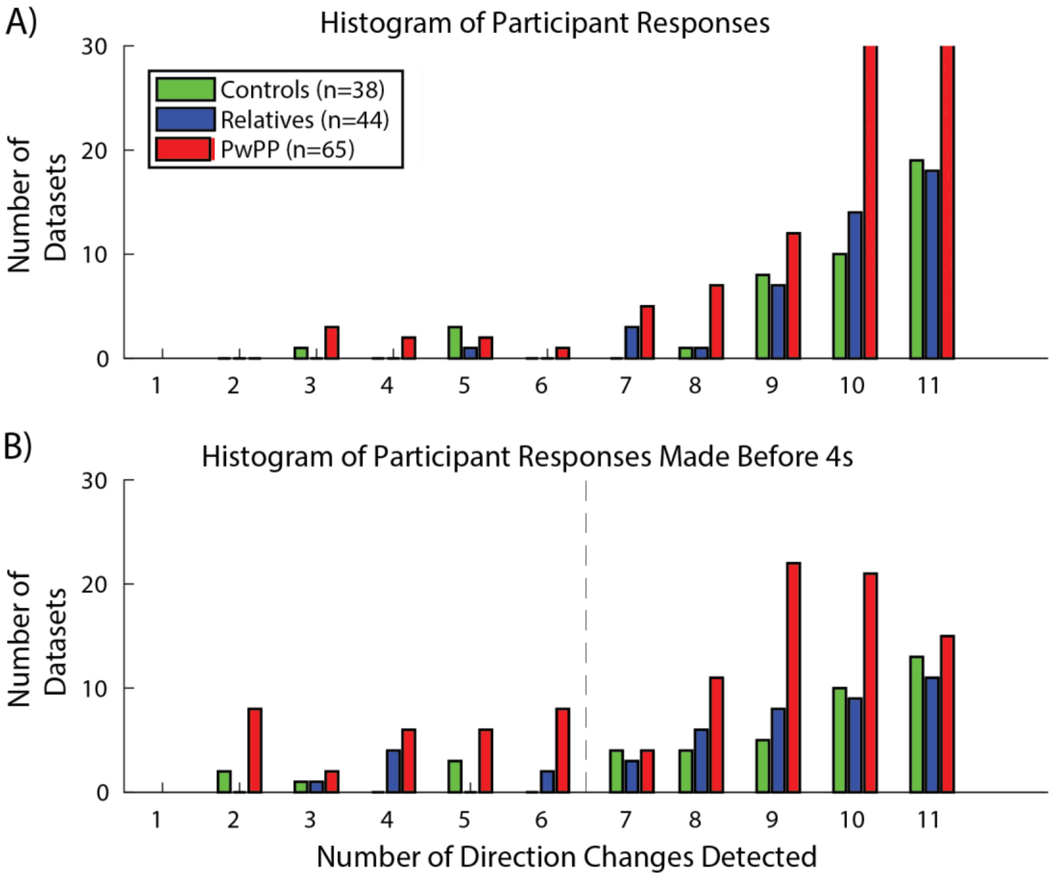

Supplemental Figure 5. Distribution of the number of responses made across all participants. Plotted in panel A are the number of datasets where participants responded correctly to the corresponding number of physical switches. Plotted in panel B are the same data thresholded by a response time of 4 seconds. The colored bars represent the three groups and the vertical dashed line represents a cutoff of 63.4% or that the participant responded to at least 7/11 physical switches.

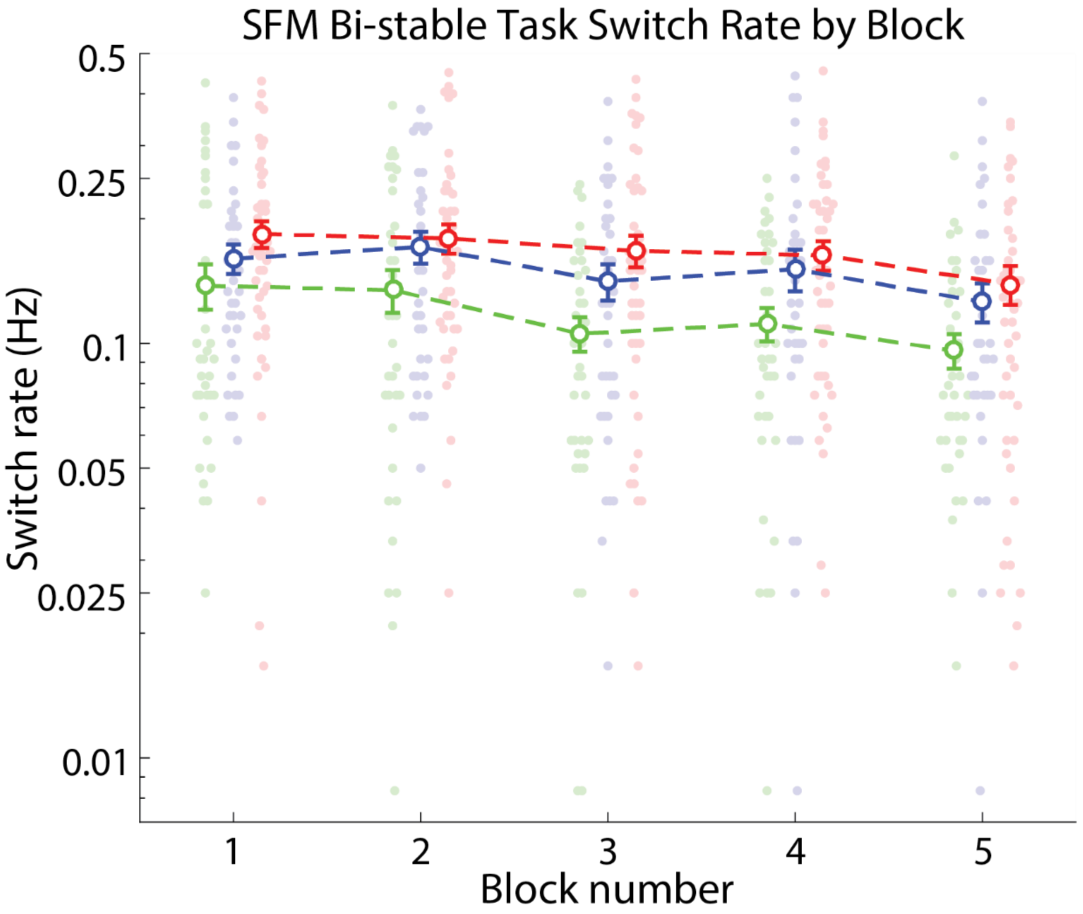

Supplemental Figure 6. Switch rate for each group over time (block). Plotted are the average switch rates during each block for all three groups (controls (n=37) – green, relatives (n=37) – blue, PwPP (n=48) – red). Error bars represent the standard error of the mean.

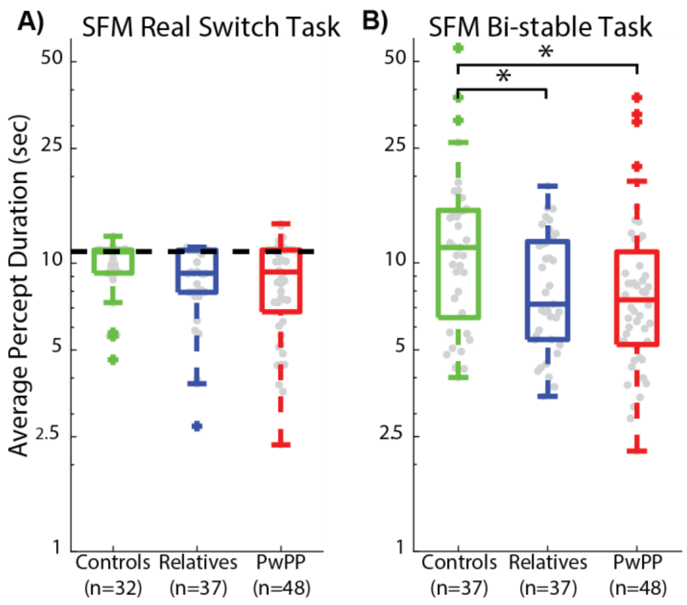

Supplemental Figure 7. Average percept duration for the three groups in (A) the real switch and (B) bi-stable tasks. Bi-stable percept durations (B) were significantly higher among PwPP vs controls as well as among PwPP vs relatives, using post-hoc chi-squared tests (*). Shown are box plots displaying median, 25%-75% quartiles, and 1.5 x the interquartile range for the three groups (controls – green, relatives – blue, PwPP – red). Outliers are shown overlaid on crosses. Dashed line in A shows average percept duration for physical stimulus changes in the real switch task.

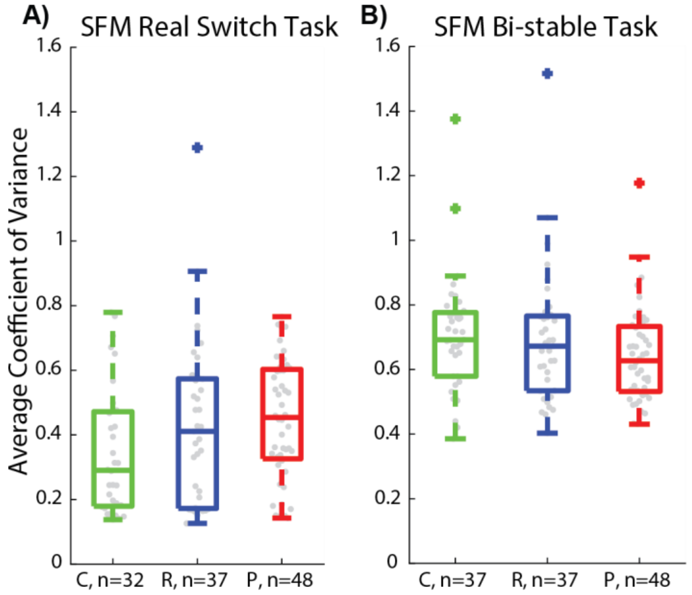

Supplemental Figure 8. Average coefficient of variance (*SD* divided by the mean) for percept durations in both the (A) real switch and (B) bi-stable tasks. Shown are box plots displaying median, 25%-75% quartiles, and 1.5 x the interquartile range for the three groups (controls – green, relatives – blue, PwPP – red). Outliers are shown overlaid on crosses.

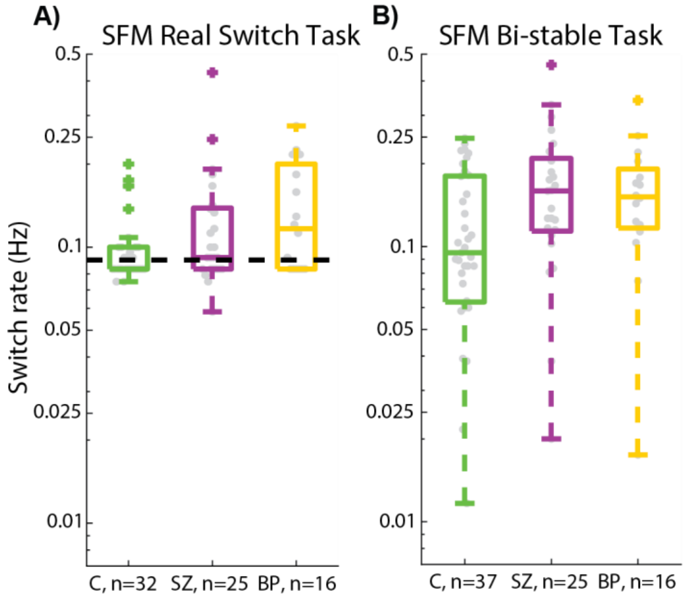

Supplemental Figure 9. Average switch rate for the control, bipolar, and schizophrenia groups in (A) the real switch and (B) bi-stable tasks. Shown are box plots displaying median, 25%-75% quartiles, and 1.5 x the interquartile range for the three groups (controls (n=37) – green, schizophrenia (n=25) – purple, bipolar (n=16) – yellow). Outliers are shown overlaid on crosses.

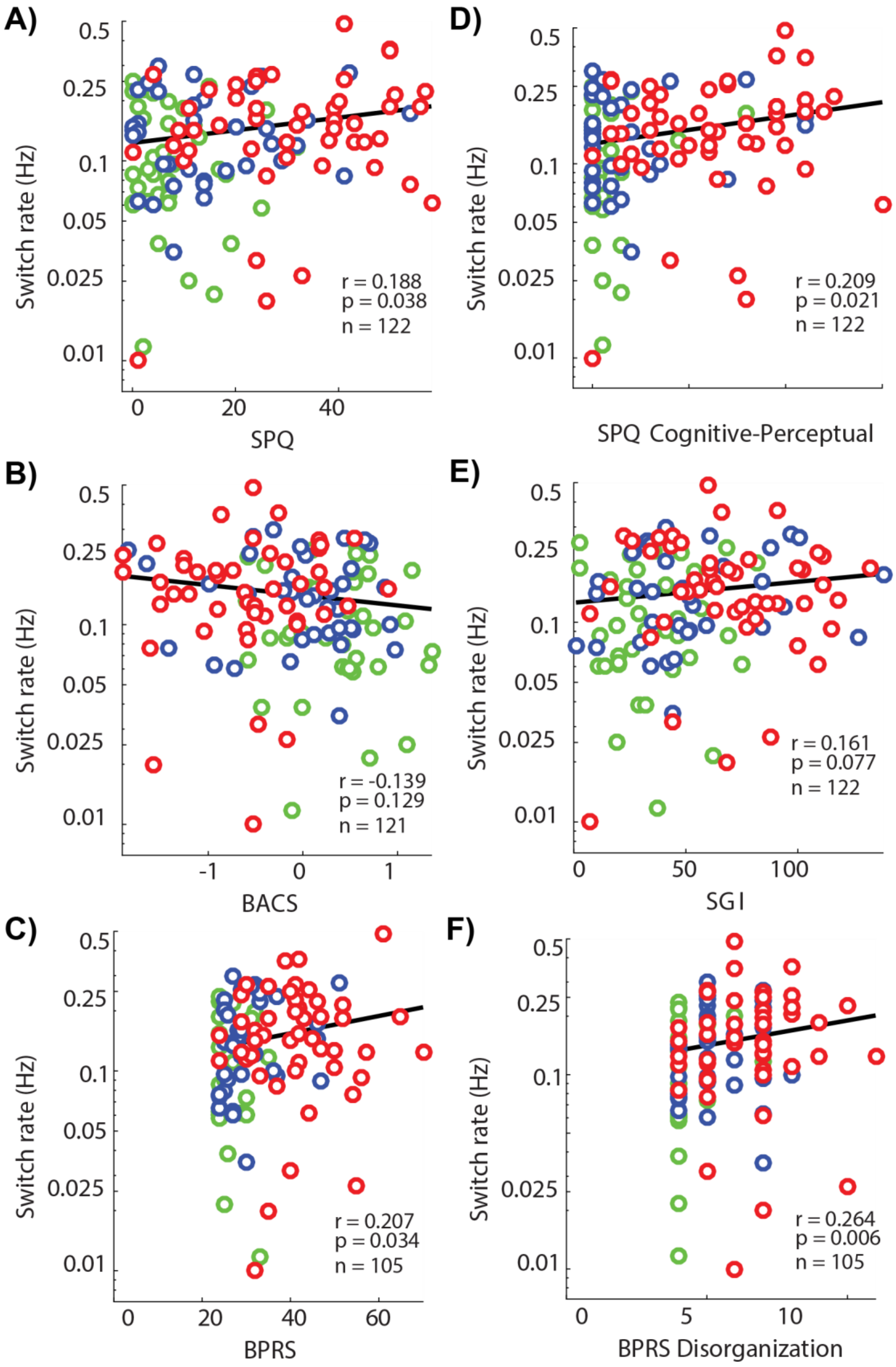
A)
B)
C)
D)
E)
F)
Switch rate (Hz)
0.5
0.25
0.1
0.05
0.025
0.01
r = 0.188
p = 0.038
n = 122
SPQ
r = -0.139
p = 0.129
n = 121
BACS
r = 0.207
p = 0.034
n = 105
BPRS
r = 0.209
p = 0.021
n = 122
SPQ Cognitive-Perceptual
r = 0.161
p = 0.077
n = 122
SGI
r = 0.264
p = 0.006
n = 105
BPRS Disorganization

Supplemental Figure 10. Bi-stable switch rate correlations with measures of psychotic symptomology. Relationship between switch rate and 6 measures of psychotic symptomology: (A) SPQ, (B) BACS, (C) BPRS, (D) SPQ Cognitive-Perceptual Factor, (E) SGI, (F) BPRS Disorganization Factor. Plotted points represent individual participants broken down by color for different groups (controls (n-31) – green, relatives (n-37) – blue, PwPP (n=48) – red). Y-axes show bi-stable switch rates (Hz) and x-axes show clinical test scores. R-values indicate Spearman correlation with retest data excluded. P-values are not corrected for multiple comparisons.

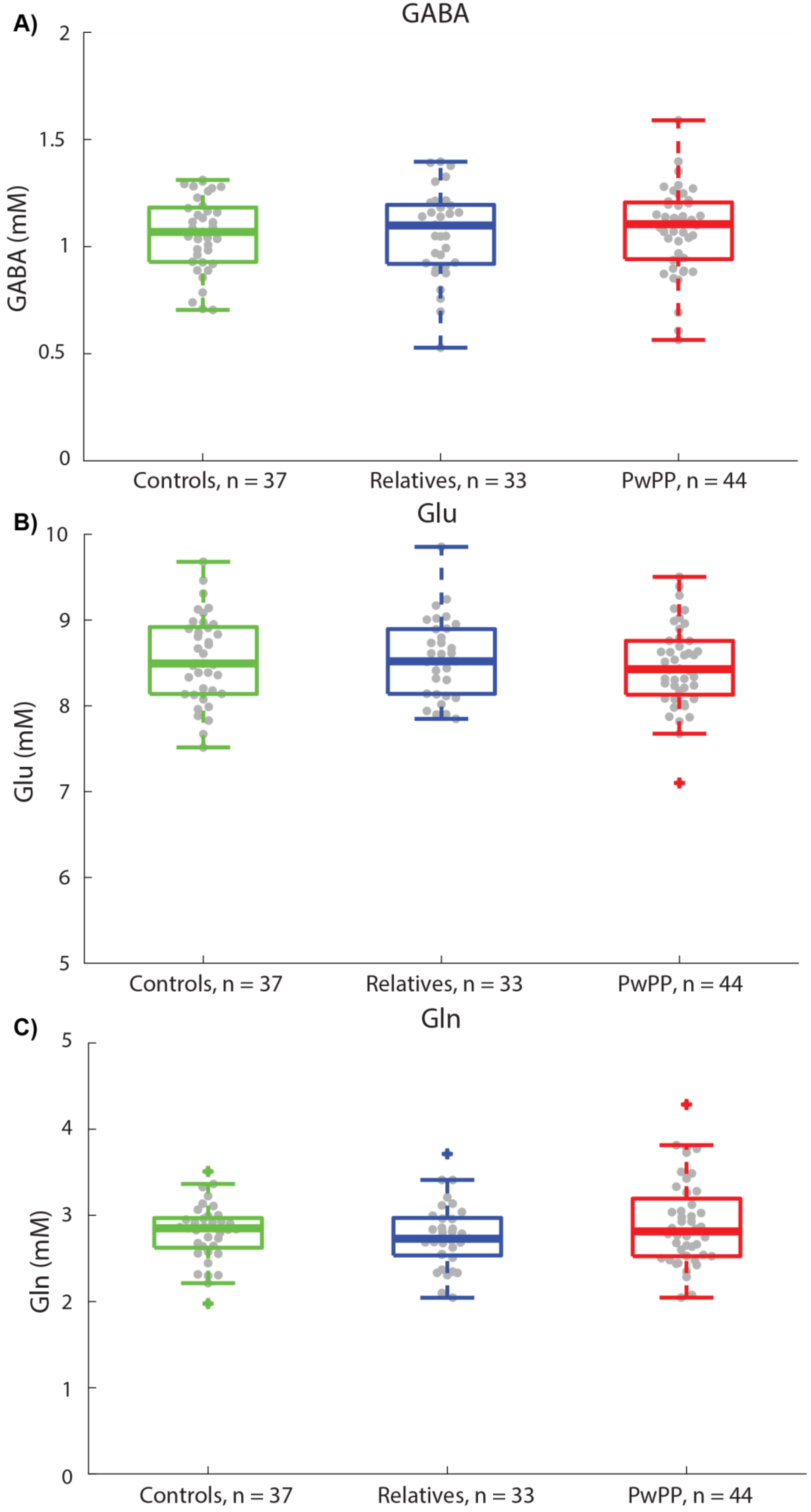

A)
GABA
GABA (mM)
0
0.5
1
1.5
2
Controls, n = 37
Relatives, n = 33
PwPP, n = 44
B)
Glu
Glu (mM)
5
6
7
8
9
10
Controls, n = 37
Relatives, n = 33
PwPP, n = 44
C)
Gln
Gln (mM)
0
1
2
3
4
5
Controls, n = 37
Relatives, n = 33
PwPP, n = 44

Supplemental Figure 11. MRS metabolite concentrations in the three groups. Average metabolite concentration (mM) for the three selected neurochemicals: (A) GABA, (B) glutamate (Glu) (B), and (C) glutamine (Gln). Shown are box plots displaying median, 25%-75% quartiles, and 1.5 x the interquartile range for the three groups (controls – green, relatives – blue, PwPP– red). Outliers are shown overlaid on crosses.

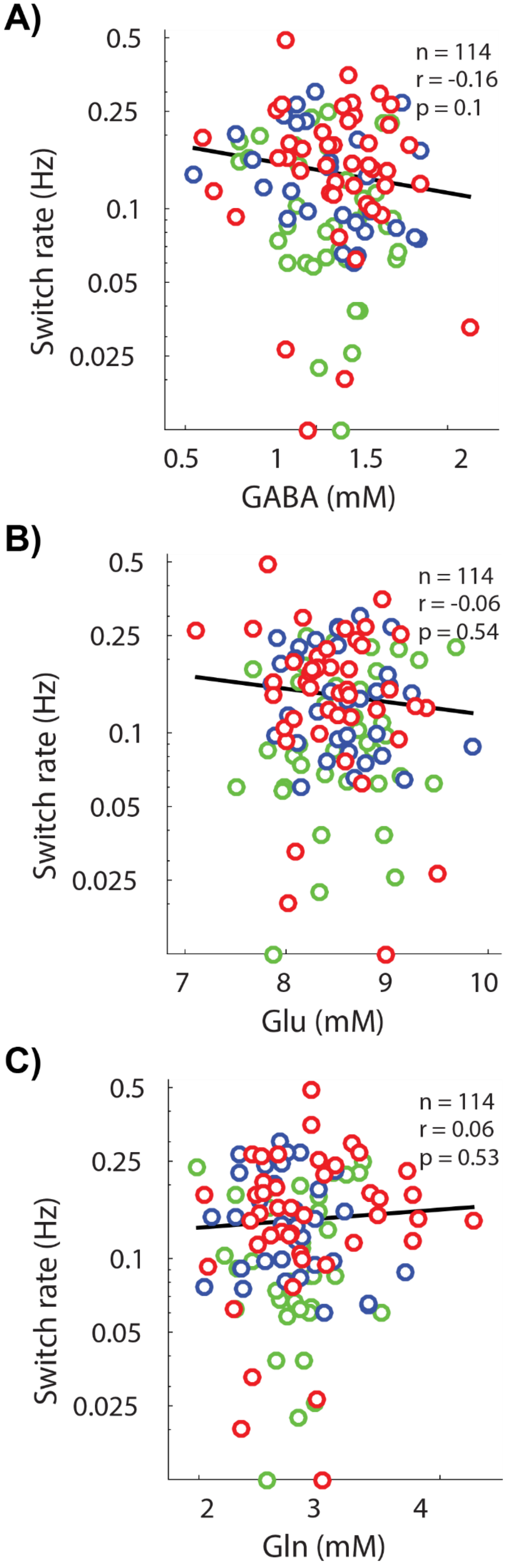
A)
n = 114
r = -0.16
p = 0.1
Switch rate (Hz)
0.5
0.25
0.1
0.05
0.025
0.5
1
1.5
2
GABA (mM)
B)
n = 114
r = -0.06
p = 0.54
Switch rate (Hz)
7
8
9
10
Glu (mM)
C)
n = 114
r = 0.06
p = 0.53
Switch rate (Hz)
2
3
4
Gln (mM)

Supplemental Figure 12. Bi-stable switch rate correlations with neurochemical concentrations. Correlations between switch rate (Hz) and concentrations of three metabolites: (A) GABA, (B) glutamate, (C) and glutamine. Plotted on the y-axis are bi-stable switch rates (Hz) and on the x-axis neurochemical concentrations (mM). Plotted points represent individual participants broken down by color for different groups (controls (n=37) – green, relatives (n=33) – blue, PwPP (n=44) – red)

Supplemental tables

Supplemental Table 1. Subject group demographics including only subjects from the bi-stable analysis, after exclusion. Data are presented as mean (standard deviation), unless otherwise specified. Estimated IQ was measured using the Wechsler Adult Intelligence Scale (WAIS-IV; Wechsler, 2008). Visual acuity was measured using a Snellen visual acuity test (Snellen, 1862). The Snellen fraction is reported (e.g., 1 indicates 20/20 vision). The statistics column shows the test statistics and *p*-values calculated across the three groups for each measure. Highlighted entries indicate significant group differences at $p < 0.05$. For any measure in which normality and / or homogeneity of variance were not observed, non-parametric Kruskal-Wallis tests (X^2) were used in place of ANOVAs (F).

	**Healthy Controls** (n = 37)	**First-degree Relatives** (n = 37)	**PwPP** (n = 48)	**Statistics**
Age (years)	38 (12.27)	43.73 (12.5)	39.56 (12.38)	$F_{(2,119)} = 2.14$<br>$p = 0.12$
Sex assigned at birth (% female)	59%	65%	48%	$X^2_{(2)} = 2.62$<br>$p = 0.269$
Years of education	16.3 (2.17)	15.5 (1.8)	14.5 (2.52)	**$F_{(2,119)} = 7.03$**<br>**$p < 0.01$**
Estimated IQ	107 (9.6)	104 (9.7)	97 (11.1)	**$F_{(2,119)} = 12.16$**<br>**$p < 0.001$**
Visual acuity (Snellen fraction)	0.975 (0.28)	0.92 (0.264)	0.96 (0.599)	$F_{(2,80)} = 0.095$<br>$p = 0.91$
